# Is there a relationship between length of resection and lymph-node ratio in colorectal cancer?

**DOI:** 10.1093/gastro/goz066

**Published:** 2020-12-28

**Authors:** Antonio Zanghì, Andrea Cavallaro, Emanuele Lo Menzo, Serena Curella Botta, Salvatore Lo Bianco, Maria Di Vita, Francesco Cardì, Alessandro Cappellani

**Affiliations:** 1 General and Breast Surgery Unit, Department of Surgery, University of Catania, Catania, Italy; 2 Department of General Surgery, Digestive Disease Institute, Cleveland Clinic Florida, Weston, FL, USA

**Keywords:** colorectal cancer, lymph-node ratio, resection length, prognostic value

## Abstract

**Background:**

The prognosis of colorectal cancer depends on the number of positive lymph nodes (LN+) and the total number of lymph nodes resected (rLN). This represents the lymph-node ratio (LNR). The aim of our study is to assess how the length of the resected specimen (RL) influences the prognostic values of the LNR.

**Methods:**

We conducted a retrospective study of all the patients operated on for colorectal cancer from 2000 to 2015 at our institution. Pathology details were analysed. The total number of rLN, the number of LN+, and the LNR were calculated and measured against the RL. The receiver-operating characteristic (ROC) curve of patients with LN+ was calculated.

**Results:**

Of the 670 patients included in our study, 337 were men (50.3%) and the mean age was 69.2 years. The correlation with prognosis of the LNR is greater than that of the LNR adjusted to RL (LNR/RL), both in subjects with positive nodes (*n *=* *312) and in all cases (*n *=* *670). The LNR presents a higher prognostic value than LNR/RL and RL in patients with LN+ except for metastatic recurrence, for which the predictive value appears slightly higher for LNR/RL. The statistical significance of the maximal divergence in Kaplan–Meier survival plots was demonstrated for the LNR (*P *=* *0.043), not for LNR/RL (*P *=* *0.373) and RL alone (*P *=* *0.314).

**Conclusion:**

An increase in RL causes an increase in the number of harvested lymph nodes without affecting the number of LN+, thus representing a confounding factor that could alter the prognostic value of the LNR. Prospective larger-scale studies are needed to confirm these findings.

## Introduction

In several countries, colorectal cancer (CRC) represents the second neoplasia by incidence in both men and women. According to the Italian Cancer Registries’ report of 2017, the 5-year survival rate for CRC was 66% [1]. The most important predictors of long-term survival reported were distant metastasis and regional lymph-nodes status. The prognosis, however, depends not only on the number of positive lymph nodes collected in the sample (LN+), but also on the number of lymph nodes resected (rLN) independently of their positivity [2, 3]. In fact, a higher number of rLN positively affects survival, while an increase in the number of LN+ among those collected has negative effects on long-term survival, as it is directly linked to more advanced disease [4, 5]. Therefore, the assessment of the lymph-node status represents a key point for the choice of the correct surgical and chemotherapeutic treatment. 

Based on international scientific evidence, the Italian Association of Medical Oncology (AIOM) guidelines recommend the collection of ***≥***12 lymph nodes in all CRC patients, with the exception of cases that received neoadjuvant chemotherapy or radiotherapy [6–8]. The need to take into account both the rLN and the LN+ has led to prognostic use of the lymph-node ratio (LNR), which has been recognized by numerous clinical studies as an independent prognostic factor [5, 9–16].

The number of rLN is obviously dependent on numerous factors, including the length of the resected surgical specimen. However, there are as yet no established guidelines on the length of the surgical specimen; consequently, the sample length falls within a very wide range of variability and depends on the patient characteristics, the site, the margins, and the choices of the surgeon. The aim of our study is to recognize how the resection length (RL) affects the prognostic value of the LNR. In addition, we will investigate whether the correct relationship between the LNR and RL has a greater prognostic value than the unrelated data.

## Patients and methods

### Study subjects

A retrospective analysis of patients treated for CRC from January 2000 to December 2015 at the Department of Surgery of the Policlinico of Catania, Italy was conducted. All patients treated for CRC with curative intent, and with pre- and post-operative and follow-up data available, were included in the study, giving particular attention to anatomopathological data concerning TNM staging, lymph-node status, and the length of resection. Patients in whom neoadjuvant therapy was administrated were excluded from this study. The patient evaluation included clinical history, preoperative staging of the disease (ultrasound, computed tomography, magnetic resonance imaging, positron emission tomography, and scintigraphy in some cases), associated with appropriate blood tests (CA19-9 and CEA). All the procedures aimed to achieve a microscopic free distal margin of ***≥***5 cm and a number of lymph nodes ≥12 for proper pathological staging. Several surgeons performed the resections, although they were all experienced and with similar skill levels.

### Data collection and outcome measurement

The data necessary for the execution of this study were obtained from the Cancer Registry of our institution and the hospital archive, and subsequently were integrated with the data contained in the medical records and in the reports of the anatomopathological examinations. The surgical specimens were analysed by two local pathology laboratories. With the exception of cases in which the tumor macroscopic distance was critical, the specimen was measured in the pathology department after formalin fixation.

Data regarding follow-up, and in particular the relapse-free survival (RFS) and overall survival (OS), were obtained from the medical records or the hospital information system and, when not directly available, they were collected through a telephone interview conducted by a single individual and with a standardized telephone script, in an attempt to minimize any bias related to the type of investigation.

The LNR was calculated, as conventionally defined, by dividing the number of LN+ by the rLN. The LNR was then adapted to the value of RL, in a relationship defined as the ratio of LN+ to rLN per centimeter of resected bowel, which can be simplified to LNR/RL. To verify whether the RL improves the prognostic value of the LNR, we first reported the LNR with the data available at the follow-up, then also the LNR adapted to the RL, and finally only with the RL.

### Statistical analysis

The analysis was performed using the software package Statsoft STATISTICA v.10 and R Software v.3.2.5. The statistical-significance limit was set to a *P*-value <0.05 for all analyses. The Shapiro–Wilk test was performed to evaluate normality. The baseline characteristics were presented as mean and standard deviation, or median and interquartile range (IQR). The correlation between the LNR models and the dichotomous outcomes was evaluated using the Spearman Rho coefficient (*r*), since a linear relationship between the LNR models and the outcomes is not expected. For the estimation of sensitivity and specificity in predicting long-term outcomes, the receiver-operating characteristic (ROC) curves were used that correlated the three variables (LNR, LNR/RL, RL) to the presence of metastases and to the event of death or recurrence. Lastly, a plot of the Kaplan–Meier estimator was obtained with the aim to evaluate the value of the OS and RFS function related to the N-state, LNR, and LNR/RL variables.

## Results

Of the 670 patients included in our study, 337 were men (50.3%). The mean age was 69.2 ± 11.3 years. The median number of rLN was 17 (IQR, 10–25). The data concerning rLN, LN+, and RL were evaluated over the totality of the 670 patients analysed. However, regarding RFS and OS, it was possible to recover full confirmed data only from 285 of the 312 LN+ patients (91.3%) and 326 of 358 LN– patients (91.1%). The comparison on sensitivity and specificity of the LNR, LNR/RL, and RL was performed only on those patients whose data were fully retrieved.

### Lymph-node status

Of all patients, 312 (46.6%) had LN+, identified during post-operative anatomopathological analysis. The median number of LN+ was 0 (IQR, 0–2) and the median LNR was 0 (IQR, 0–0.14). The anatomopathological analysis allowed the RL to be estimated, which has a mean value of 30.66 ± 16.47 cm. Considering data in only LN+ patients, the median number of LN+ was 2 (IQR, 1–5), and the median LNR was 0.167 (IQR, 0.08–0.38). The RL has a mean value of 30.50 ± 15.19 cm (Table 1).

**Table 1.** Anatomopathological parameters of study population

Parameter	Total patients (*n* = 670)	Patients with positive nodes (*n* = 312)
No. of positive lymph nodes	0 (0–2)	2 (1–5)
Lymph-node ratio	0 (0–0.14)	0.167 (0.08–0.38)
Resection length, cm	30.66 ± 16.47	30.50 ± 15.19

Values were presented as mean ± standard deviation or median (interquartile range).

### Correlation between the LNR, LNR/RL, RL, and prognosis

The correlations between the LNR, LNR/RL, RL, and prognosis are shown in Table 2. The correlations, evaluated using Spearman’s Rho coefficient, were significant for a good percentage of cases. In general, however, the significance of the LNR was greater than that of the LNR adjusted to RL, both in all cases and in subjects with LN+. In the group comprising the totality of patients, only the LNR is significant in all cases except in the case of recurrence, in which both the LNR and LNR/RL did not demonstrate statistical significance. In the group with LN+, instead, both indicators showed significance in all cases, with the exception of the recurrence event in relation to the LNR. So the LNR alone would not be predictive of recurrence in either LN+ patients, whereas LNR/RL is weakly statistically significant for distal metastasis, recurrence, and death in LN+ patients. The single RL factor did not show statistical significance in any of the cases.

**Table 2.** Correlations (*r*) between lymph-node ratio (LNR), resection length (RL), LNR/RL, and long-term outcomes evaluated by using Spearman’s Rho coefficient

Outcome	Total patients (*n* = 670)			Patients with positive nodes (*n* = 312)		
	LNR	LNR/RL	RL	LNR	LNR/RL	RL
Death	0.425^*^	0.413^*^	0.018	0.294^*^	0.257^*^	0.023
Recurrence	0.035	0.036	0.048	0.073	0.073^*^	0.068
Distal metastasis	0.246^*^	0.236^*^	0.025	0.141^*^	0.108^*^	0.044

* *P *<* *0.05.

### ROC-curves analysis

**Figure 1.**
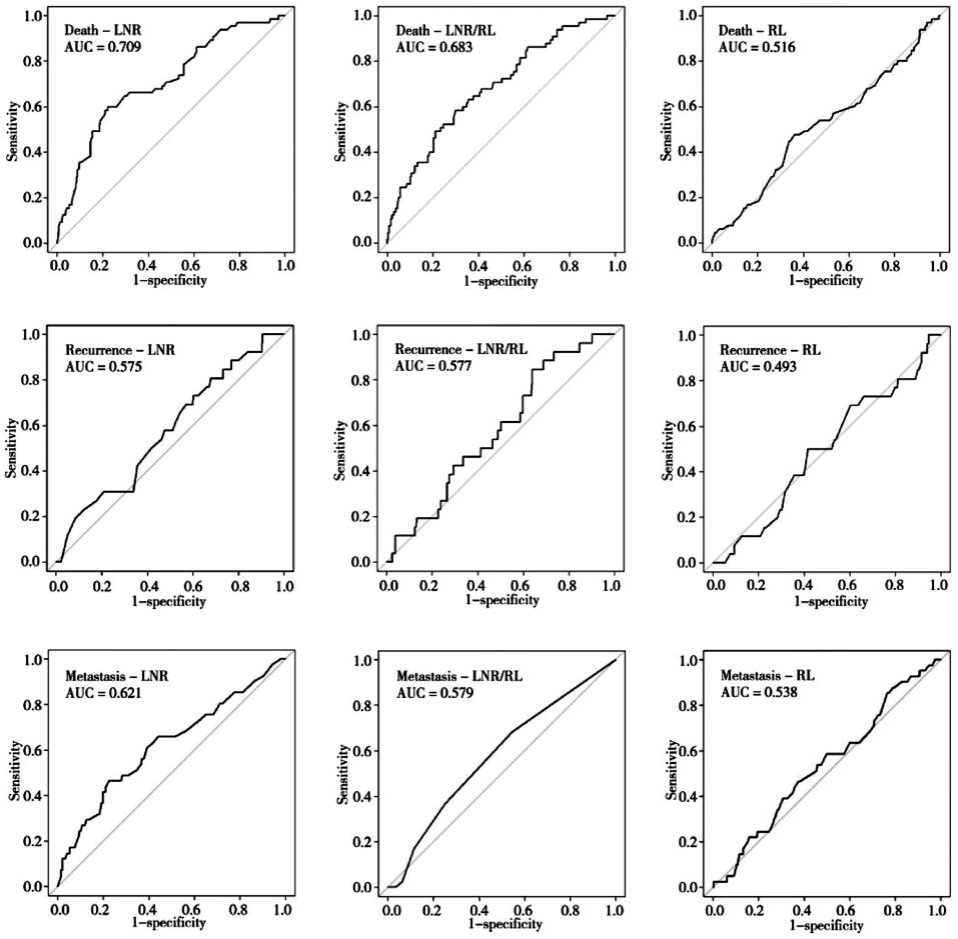
The receiver-operating characteristic (ROC) curve for lymph-node ratio (LNR), resection length (RL), and LNR/RL in predicting long-term outcomes of colorectal-cancer patients with positive lymph nodes. The area under the curve (AUC) represents the area between the reference line and the specific curve, where a larger area denotes a higher predictive value.

The ROC curves were calculated for the population of LN+ patients (*n *=* *312) representing the population in which the ratio of our interest (LNR/RL) has potentially the highest prognostic value (cases with LN– would produce a ratio of 0 in both the LNR and LNR/RL, thus making redundant values). The area under the curve (AUC) demonstrated that the LNR presented a higher prognostic value than the other two indices in LN+ cases except for recurrence, for which the predictive value of LNR/RL appeared slightly higher (Figure 1). The prognostic value obtained for just the RL was instead the lowest among those analysed, denoting a less reliable predictive value and therefore the absence of correlation with the prognosis.

### Kaplan–Meier plots of OS and RFS

**Figure 2.**
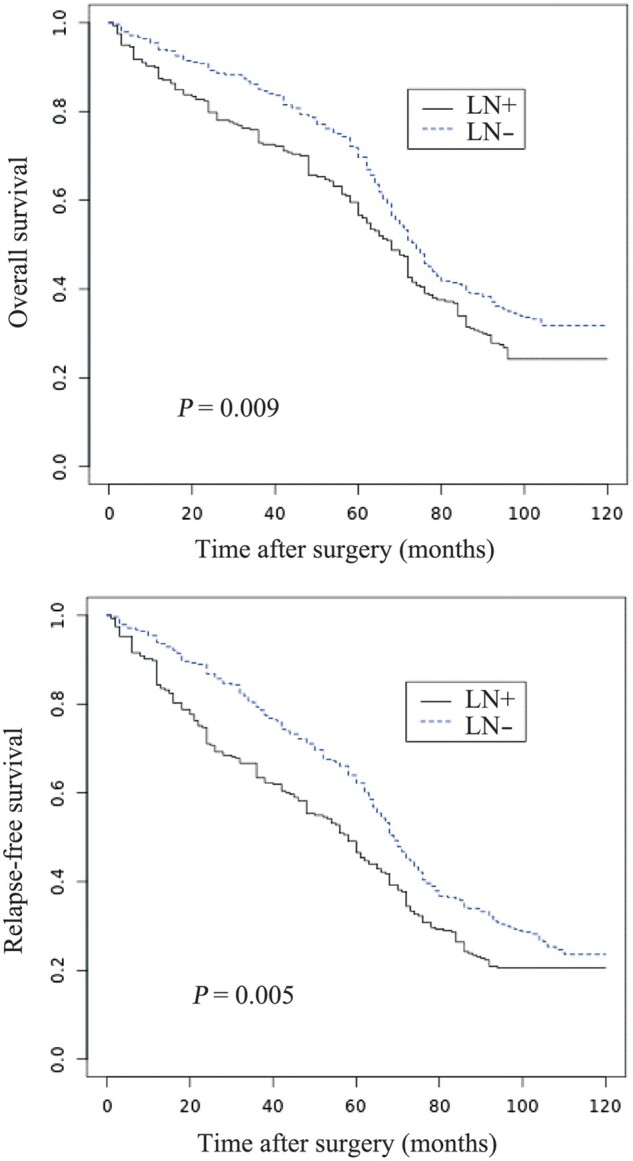
Overall survival (A) and relapse-free survival (B) in colorectal-cancer patients related to lymph-node status.

**Figure 3.**
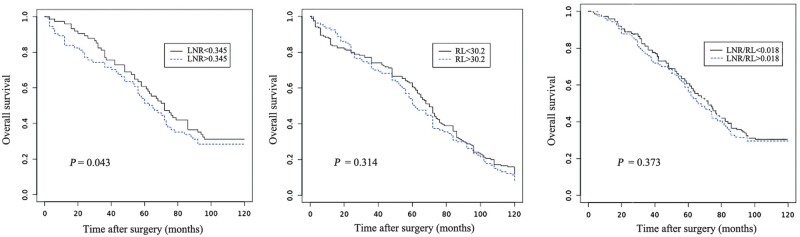
Overall survival above and under the ideal threshold of lymph-node ratio (LNR; threshold, 0.345) (A), resection length (RL; threshold, 30.2 cm) (B), and LNR adjusted to RL (LNR/RL; threshold, 0.018) (C) in colorectal-cancer patients with positive lymph nodes.

A plot of the Kaplan–Meier estimator was obtained with the aim to evaluate OS and RFS related to the LN state, LNR, and LNR/RL variables. The 5-year OS was 59.7% in the LN+ patients and 72.2% in the LN– patients. The 5-year RFS in the LN+ and LN– patients was 48.8% and 63.6%, respectively (Figure 2). We calculated, among the LN+ patients, the ideal threshold of the LNR value (0.345) above and under which OS demonstrated maximal divergence (*P *=* *0.043). Instead, the LNR/RL (*P *=* *0.373) and RL alone (*P *=* *0.314) demonstrated themselves as unreliable predictive indicators with absence of correlation with the prognosis (Figure 3).

## Discussion

The ability to accurately predict survival and the risk of recurrence has been a primary focus of the oncologists. The number of LN+ plays an important role in the TNM system. But the N-stage assessment is easily influenced by the extent of lymph-node dissection, length of the surgical specimen, surgeon’s technique, and the thoroughness of the pathologist. It has been shown that ≥12 lymph nodes need to be evaluated in order to accurately assign the ‘true’ nodal status of CRC patients. The proposal of a lymph-node ‘threshold’ aims to prevent inadequate sampling and under-staging, as worse survival can occur due to stage migration and no administration of adjuvant chemotherapy. Some investigators have questioned the use of 12 lymph nodes examined as an arbitrary cut-off value. A higher number of lymph nodes examined is associated with survival—a finding that cannot be attributable to under-staging only. Differences in quality of care, patient characteristics, and tumor biology may explain these findings. In fact, different studies show that lymph-node diffusion beyond the 10-cm margin is generally rare, with a probability of positive results ranging from 0% to 4%. Based on these data, the extent of the resection would be unjustified, also involving the risk of a dilution of the ratio between LN+ and rLN. However, we know that longer lymph-node deliveries correspond to longer resection, so the RL is a factor that could correlate and increase the predictive value of the LNR alone.

The LNR, defined as the ratio between the number of LN+ and the total number of rLN, has gained some consensus over the years as a more reliable and accurate prognostic indicator than the number of LN+ alone. It has been established that the LR influences the number of lymph nodes collected. In fact, longer resections are linked to a greater number of lymph nodes collected, which can lead to underestimating the LNR value. The result is an increased possibility of recognizing LN+ for neoplastic infiltration but, at the same time, a ‘dilution effect’ on the LN+ and therefore a reduction in the LNR value.

In the last decade, several studies have demonstrated the LNR to be a superior prognostic indicator and some authors propose incorporating the LNR into the TNM staging system. The LNR is an instrument already in use, in addition to the classic staging, to stratify patients with tumors in the breast [9], stomach [10], pancreas [11], rectum [12], gallbladder [13], melanoma [14], and colon [5, 15]. All national and international guidelines recommend the evaluation of ≥12 lymph nodes to prevent false-negative samples, so the greater the number of lymph nodes collected, the lower the risk of false-negative results.

Table 3 summarizes the prognostic significance of the LNR in different studies [15–30]. De Ridder *et al*. [15] analysed 26,181 CRC patients from SEER data and the LNR appeared to be a strong independent risk factor (*P *<* *0.0001). Prognostic separation using the LNR was 31% compared with 26% using the UICC pN stage. Chin *et al*. [18] compared stage III CRC patients with an LNR of ***≤***0.4 (LNR1), 0.4–0.7 (LNR2), and >0.7 (LNR3). The 5-year disease-free survival (DFS) was higher in LNR1 than that in the other two categories. The 2-year DFS for patients with an LNR >0.7 was 0%, suggesting that this group of patients should be approached and treated as stage IV patients. They concluded that the LNR was a more precise predictor of DFS in stage III patients than the total number of positive nodes. Greenberg *et al*. [20] found that an LNR <0.13 was associated with a significant increase in OS and a strong trend toward improved DFS. Interestingly, the survival curves in stage III patients with an LNR <0.13 were similar to our stage II node-negative patients. According to Moug *et al*. [25], only patients with an LNR of 0.05–0.19 had long-time-survival benefits from adjuvant chemotherapy. Based on the LNR degree, Sugimoto *et al*. [26] proposed a new classification based on the combination of T category (T1, T2, T3, T4a, and T4b) and LNR (LNR-low and LNR-high).

**Table 3.** Summary of the cited studies

Authors	Year	No. of patients	LNR cut-off	Statistical relevance
De Ridder *et al*. [15]	2006	26,181	0.4	OS, DFS
Wang *et al*. [16]	2008	24,477	1/14, 0.25, and 0.50	OF, DFS
Priolli *et al*. [17]	2009	113	0.20	OS, DFS
Chin *et al*. [18]	2009	490	<0.4, 0.4–0.7, >0.7	DFS
Hong *et al*. [19]	2010	130	0.1638	DFS
Greenberg *et al*. [20]	2011	65	0.13	OS, DFS
Tuna *et al*. [21]	2011	125	0.2	DFS
Wang *et al*. [22]	2012	256	<0.11, 0.11–0.39, >0.39	DFS
Lu *et al*. [23]	2013	612	0.17	OS, DFS
Sabbagh *et al*. [24]	2014	178	0.1	OS, DFS
Moug *et al*. [25]	2014	673	<0.05, 0.05–0.19, 0.20–0.39, 0.40–1.00	OS, DFS
Sugimoto *et al*. [26]	2015	4,172	0.18	OS
Mohan *et al*. [27]	2017	402	0.27	OS
Jakob *et al*. [28]	2018	85	0.125	None
Amri *et al*. [29]	2016	1,039	LNR/RL	None
Gleisner *et al*. [30]	2013	154,208	N-score	OS, DFS

LNR, lymph-node ratio; LNR/RL, lymph-node ratio per length of resection; OS, overall survival; DFS, disease-free survival.

However, some authors expressed some criticism to the role of the LNR. Mohan *et al*. [27] compared the LNR1/LNR2 ratio with N1/N2 to determine whether, for a given specificity, the LNR had a better sensitivity. Use of the LNR resulted in a small improvement in sensitivity, but this did not reach statistical significance. Therefore, the authors conclude that, while the LNR is predictive of OS, it does not offer a clinically significant advantage over the current N1/N2 classification. Also, Jakob *et al*. [28] agreed that the LNR was inferior to the pN category in predicting recurrence and survival for stage III colon-cancer patients with a high number of analysed lymph nodes. Amri *et al*. [29] studied the role of the RL and whether an increase in the RL may result in a dilution of the fraction of positive nodes through a disproportionate increase in harvested nodes, which impacts the LNR in LN+ cases and may undermine its positive predictive value. Their analysis shows that the RL does not significantly impact the prognostic value of the LNR in colon cancer.

A challenging question emerges: what does the LNR serve as a marker for? A patient staged with a lower LNR underwent better surgical and pathological management, which could reflect the biological nature of the primary colonic tumor either at a genetic level or at a cellular level with poor prognostic pathological features (including microscopic spread or extramural vascular invasion) being absent. Assessment of the relationship between the LNR and local and distant recurrence provides supporting evidence for the heterogeneity of patients with node-positive colon cancer that has potential modifications in the prescription of adjuvant chemotherapy [25].

As previously described in the literature, our study shows that the LNR is an important prognostic element. However, the RL, affecting the total number of lymph nodes harvested, potentially affected the LNR. As stated previously, the greater the number of lymph nodes collected, the better the patient outcome. In this study, the ROC curves clearly demonstrate that sensitivity and specificity are maintained in almost all the cases, higher for the LNR than for the LNR/RL, as expected. This means that the isolated RL is not linked to a benefit in survival, based on the low AUC found. Hence, although it has been established that only the RL does not have a prognostic value and that the association between the LNR and the RL reduces the specificity and sensitivity, while maintaining the significance higher for the LNR only, it is unclear whether to prefer a short resection to a longer resection. Of course, the RL must be such as to allow margins free from disease of ≥5 cm and there the collection of ≥12 lymph nodes must be allowed. A statistical significance of maximal divergence in Kaplan–Meier survival plots was demonstrated for the LNR, but not for LNR/RL and the RL alone. LNR/RL and the RL alone demonstrated themselves to be unreliable predictive indicators without correlation with the prognosis.

The limitations of our study are related to the intrinsic nature of a retrospective review. Also, because of the lack of some available data, the comparison of sensitivity and specificity was done on a smaller population, introducing potential bias.

In conclusion, our study confirms that an increase in the RL causes an increase in the number of lymph nodes, without affecting the number of LN+, thus representing a confounding factor that could alter the prognostic value of the LNR. However, the RL, isolated from other values, is not a reliable indicator for patient outcome. The LNR, instead, maintains a high prognostic value, which is useful for therapeutic purposes.

## Authors’ contributions

A.Z., A.C., S.C.B., S.L.B., M.D.V., F.C., E.L., and A.C. designed the project; A.C., S.C.B., S.L.B., M.D.V., and F.C. collected the data; A.Z., A.C., S.C.B., S.L.B., M.D.V., F.C., and A.C. analysed and interpreted the data. A.Z., A.C., E.L., M.D.V., F.C., and A.C. drafted the manuscript. A.Z., M.D.V., E.L., and A.C. critically reviewed the manuscript. All authors read and approved the final manuscript.

## Funding

No funding was used for this research. 

## References

[1] Compton CC , Fielding LP, Burgart LJ et al Prognostic factors in colorectal cancer: College of American Pathologists Consensus Statement 1999. Arch Pathol Lab Med 2000;124:979–94. 10888773 10.5858/2000-124-0979-PFICC

[2] Cunningham D , Atkin W, Lenz HJ et al Colorectal cancer. Lancet 2010;375:1030–47. 20304247 10.1016/S0140-6736(10)60353-4

[3] Quasar Collaborative Group, Gray R, Barnwell J et al Adjuvant chemotherapy versus observation in patients with colorectal cancer: a randomised study. Lancet 2007;370:2020–9. 18083404 10.1016/S0140-6736(07)61866-2

[4] Le Voyer TE , Sigurdson ER, Hanlon AL et al Colon cancer survival is associated with increasing number of lymph nodes analyzed: a secondary survey of intergroup trial INT-0089. J Clin Oncol 2003;21:2912–9. 12885809 10.1200/JCO.2003.05.062

[5] Chang GJ , Rodriguez-Bigas MA, Skibber JM et al Lymph node evaluation and survival after curative resection of colon cancer: systematic review. J Natl Cancer Inst 2007;99:433–41. 17374833 10.1093/jnci/djk092

[6] Compton CC , Greene FL. The staging of colorectal cancer: 2004 and beyond. CA Cancer J Clin 2004;54:295–308. 15537574 10.3322/canjclin.54.6.295

[7] Engstrom PF , Arnoletti JP, Benson AB et al NCCN Clinical Practice Guidelines in Oncology: colon cancer. J Natl Compr Canc Netw 2009;7:778–831. 19755046 10.6004/jnccn.2009.0056

[8] Margalit O , Mamtani R, Yang YX et al Assessing the prognostic value of carcinoembryonic antigen levels in stage I and II colon cancer. Eur J Cancer 2018;94:1–5. 29502035 10.1016/j.ejca.2018.01.112

[9] Woodward WA , Vinh-Hung V, Ueno NT et al Prognostic value of nodal ratios in node-positive breast cancer. J Clin Oncol 2006;24:2910–6. 16782931 10.1200/JCO.2005.03.1526

[10] Zhou YX , Yang LP, Wang ZX et al Lymph node staging systems in patients with gastric cancer treated with D2 resection plus adjuvant chemotherapy. J Cancer 2018;9:660–6. 29556323 10.7150/jca.22016 PMC5858487

[11] Elshaer M , Gravante G, Kosmin M et al A systematic review of the prognostic value of lymph node ratio, number of positive nodes and total nodes examined in pancreatic ductal adenocarcinoma. annals 2017;99:101–6. 10.1308/rcsann.2016.0340 PMC5392844 27869496

[12] Klos CL , Bordeianou LG, Sylla P et al The prognostic value of lymph node ratio after neoadjuvant chemoradiation and rectal cancer surgery. Dis Colon Rectum 2011;54:171–5. 21228664 10.1007/DCR.0b013e3181fd677d

[13] Aoba T , Ebata T, Yokoyama Y et al Assessment of nodal status for perihilar cholangiocarcinoma: location, number, or ratio of involved nodes. Ann Surg 2013;257:718–25. 23407295 10.1097/SLA.0b013e3182822277

[14] Xing Y , Badgwell BD, Ross MI et al Lymph node ratio predicts disease-specific survival in melanoma patients. Cancer 2009;115:2505–13. 19309746 10.1002/cncr.24290 PMC2755291

[15] De Ridder M , Vinh-Hung V, Van Nieuwenhove Y et al Prognostic value of the lymph node ratio in node positive colon cancer. Gut 2006;55:1681. 10.1136/gut.2006.104117 PMC1860091 17047131

[16] Wang J , Hassett JM, Dayton MT et al Lymph node ratio: role in the staging of node-positive colon cancer. Ann Surg Oncol 2008;15:1600–8. 18327530 10.1245/s10434-007-9716-x

[17] Priolli DG , Cardinalli IA, Pereira JA et al Metastatic lymph node ratio as an independent prognostic variable in colorectal cancer: study of 113 patients. Tech Coloproctol 2009;13:113–21. 19484349 10.1007/s10151-009-0467-5

[18] Chin CC , Wang JY, Yeh CY et al Metastatic lymph node ratio is a more precise predictor of prognosis than number of lymph node metastases in stage III colon cancer. Int J Colorectal Dis 2009;24:1297–302. 19479270 10.1007/s00384-009-0738-7

[19] Hong KD , Lee SI, Moon HY. Lymph node ratio as determined by the 7th edition of the American Joint Committee on Cancer staging system predicts survival in stage III colon cancer. J Surg Oncol 2011;103:406–10. 21400524 10.1002/jso.21830

[20] Greenberg R , Itah R, Ghinea R et al Metastatic lymph node ratio (LNR) as a prognostic variable in colorectal cancer patients undergoing laparoscopic resection. Tech Coloproctol 2011;15:273–9. 21695442 10.1007/s10151-011-0701-9

[21] Tuna S , Dalkilic Calis M, Sakar B et al Prognostic significance of the metastatic lymph node ratio for survival in colon cancer. J Buon 2011;16:478–85. 22006754

[22] Wang J , Wang L, Ma J et al Regional lymph node staging and establishment of prognostic model for stage III colon cancer. Zhonghua Wei Chang Wai Ke Za Zhi 2012;15:1057–61. 23099906

[23] Lu YJ1 , Lin PC, Lin CC et al The impact of the lymph node ratio is greater than traditional lymph node status in stage III colorectal cancer patients. World J Surg 2013;37:1927–33. 23609344 10.1007/s00268-013-2051-4

[24] Sabbagh C , Mauvais F, Cosse C et al A lymph node ratio of 10% is predictive of survival in stage III colon cancer: a French regional study. Int Surg 2014;99:344–53. 25058763 10.9738/INTSURG-D-13-00052.1 PMC4114359

[25] Moug SJ , Oliphant R, Balsitis M et al The lymph node ratio optimises staging in patients with node positive colon cancer with implications for adjuvant chemotherapy. Int J Colorectal Dis 2014;29:599–604. 24648033 10.1007/s00384-014-1848-4

[26] Sugimoto K , Sakamoto K, Tomiki Y et al Proposal of new classification for stage III colon cancer based on the lymph node ratio: analysis of 4,172 patients from multi-institutional database in Japan. Ann Surg Oncol 2015;22:528–34. 25160735 10.1245/s10434-014-4015-9

[27] Mohan HM , Walsh C, Kennelly R et al The lymph node ratio does not provide additional prognostic information compared with the N1/N2 classification in stage III colon cancer. Colorectal Dis 2017;19:165–71. 27317165 10.1111/codi.13410

[28] Jakob MO , Guller U, Ochsner A et al Lymph node ratio is inferior to pN-stage in predicting outcome in colon cancer patients with high numbers of analyzed lymph nodes. BMC Surg 2018;18:81. 30285691 10.1186/s12893-018-0417-0 PMC6171184

[29] Amri R , Klos CL, Bordeianou L et al The prognostic value of lymph node ratio in colon cancer is independent of resection length. Am J Surg 2016;212:251–7. 27156798 10.1016/j.amjsurg.2015.10.037

[30] Gleisner AL , Mogal H, Dodson R et al Nodal status, number of lymph nodes examined, and lymph node ratio: what defines prognosis after resection of colon adenocarcinoma? J Am Coll Surg 2013;217:1090–100. 24045143 10.1016/j.jamcollsurg.2013.07.404

